# Polyunsaturated Fatty Acids: What is Their Role in Treatment of Psychiatric Disorders?

**DOI:** 10.3390/ijms20215257

**Published:** 2019-10-23

**Authors:** Paola Bozzatello, Paola Rocca, Emanuela Mantelli, Silvio Bellino

**Affiliations:** Department of Neuroscience, University of Turin, 10125 Turin, Italy; paola.bozzatello@unito.it (P.B.); paola.rocca@unito.it (P.R.); emanuela.mantelli@gmail.com (E.M.)

**Keywords:** polyunsaturated fatty acids, omega-3 fatty acids, psychiatric disorders, randomized controlled trials, efficacy, adverse effects

## Abstract

In the central nervous system omega-3 fatty acids modulate cell signaling and affect dopaminergic and serotonergic pathways. On this basis, a new application for omega-3 fatty acids has been proposed, concerning the treatment of several psychiatric disorders. The present article is an update of a previous systematic review and is aimed to provide a complete report of data published in the period between 1980 and 2019 on efficacy and tolerability of omega-3 fatty acids in psychiatric disorders. In July 2019, an electronic search on PUBMED, Medline and PsychINFO of all RCTs, systematic reviews and meta-analyses on omega-3 fatty acids and psychiatric disorders without any filter or MESH restriction was performed. After eligibility processes, the final number of records included in this review was 126. One hundred and two of these studies were RCTs, while 24 were reviews and meta-analyses. The role of omega-3 fatty acids was studied in schizophrenia, major depression, bipolar disorder, anxiety disorders, obsessive-compulsive disorder, post-traumatic stress disorder, attention deficit hyperactivity disorder (ADHD), autism spectrum disorders, eating disorders, substance use disorder and borderline personality disorder. The main evidence of the efficacy of omega-3 fatty acids has been obtained in treating depressive symptoms in patients with major depression and, to a lesser degree, bipolar depression. Some efficacy was also found in early phases of schizophrenia in addition to antipsychotic treatment, but not in the chronic phases of psychosis. Small beneficial effects of omega-3 fatty acids were observed in ADHD and positive results were reported in a few trials on core symptoms of borderline personality disorder. For other psychiatric disorders results are inconsistent.

## 1. Introduction

The role of polyunsaturated fatty acids (PUFAs) in human health acquired growing interest in the last decades. The most important classes of PUFAs are the omega-3 (ω-3) fatty acids, including α-linolenic acid (ALA), eicosapentaenoic acid (EPA) and docosahexaenoic acid (DHA), and omega-6 (ω-6) fatty acids, including linoleic and arachidonic acid. The beneficial proprieties of omega-3 fatty acids on inflammatory, cardiovascular and the nervous system are recognized by several investigations [1,2,3,4]. To date, the mechanisms underlying the effects of PUFAs are partially unknown. PUFAs are important components of phospholipids and cholesterol esters of the neuronal cell membrane, especially of dendritic and synaptic membranes. The rationale for the use of these new agents in psychiatric disorders stemmed from their primary action in producing modifications of the phospholipid fatty acid composition of the synaptic membrane and modulation of second messengers cascade [5,6,7]. In the brain, these agents modulate brain cell signaling, including dopaminergic and serotonergic pathways [8,9,10]. A well-balanced omega-3/omega-6 ratio is fundamental for development and functioning of the central nervous system. In particular, EPA and DHA constitute principal regulating factors of neurogenesis, cell survival and neurotransmission [2,11]. In recent years the effects of PUFAs were investigated in several psychiatric disorders. The role of EPA and DHA were studied in psychosis, major depression, bipolar disorder, anxiety disorders, obsessive-compulsive disorder, post-traumatic disorder, eating disorders, attention deficit hyperactivity disorder (ADHD), autism spectrum disorders, substance abuse and borderline personality disorder. The present review is an update of our previous systematic review [10] aimed to provide a complete report of the evidence from data published in the period ranging between 1980 and 2019 on efficacy and tolerability of omega-3 fatty acids in psychiatric disorders. The objective is to establish whether data collected in trials of PUFAs in the treatment of psychiatric disorders, particularly those collected in the last years, provide evidence to support their indications in specific diagnoses.

## 2. Methods

In July 2019, an electronic search on PUBMED, Medline and PsychINFO of all RCTs on omega-3 fatty acids and psychiatric disorders without any filter or MESH restriction was performed, using the following search string: “omega-3 fatty acids” and “psychiatric disorders” or “polyunsaturated fatty acids” and “psychiatric disorders” or “eicosapentaenoic acid” and “psychiatric disorders” or “docosaexaenoic acid” and “psychiatric disorders” or “ ὰ linolenic acid” and “psychiatric disorders” or “higly unsaturated fatty acids” and “psychiatric disorders” or “omega-3 fatty acids” and “schizophrenia” and “psychosis” and “depression” and “bipolar disorder” and “anxiety” and “post-traumatic disorder” and “attention deficit hyperactivity disorder” and “autism” and “eating disorders” and “impulsivity” and “personality disorders” and “substance disorders”. This string guaranteed a highly sensitive search, limiting the spelling selection of published indexed works. This logical connective led to a highly specific search that selected mostly works on the topic. We included controlled trials, meta-analyses and reviews published until July 2019. Publications had to concern efficacy and tolerability of omega-3 fatty acids in mental disorders as the principal issue. Publications written in a language other than English were excluded.

## 3. Results

**Figure 1:**
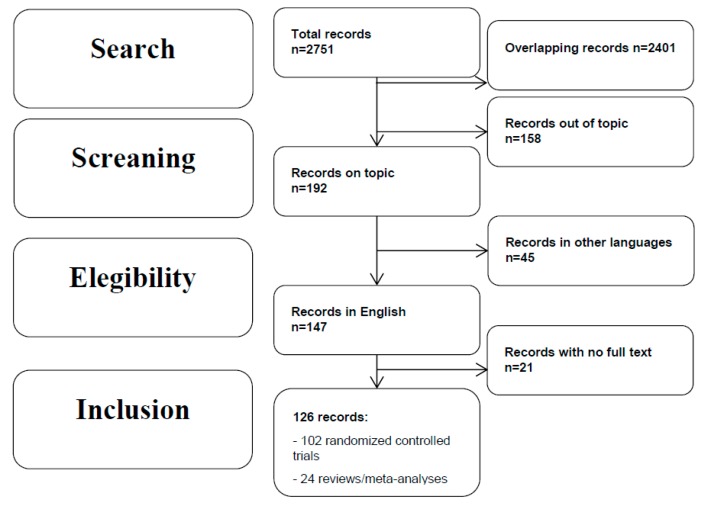
Literature search flowchart.

The search provided 2751 records. Eligibility status for all retrieved articles was determined in two stages. First, all studies were screened based upon title and abstract. The overlapping studies (2401) were excluded. Second, papers passing the initial title and abstract screen (350) were reviewed based upon the full manuscript. 158 were excluded because they did not fit the objective of the review, 45 because they were not written in English and 21 for the lack of the complete manuscript. The final number of records included was 126 (see the flow chart; Figure 1). Among these 102 were RCTs, while 24 were reviews/meta-analyses. The predominant ethnicity was Caucasian. Drop-out rates were acceptable (<20%). The majority of studies had a retention ≥ 80%.

## 4. Discussion

### 4.1. Omega-3 Fatty Acids in Schizophrenia

In both chronic and non-medicated first episode patients with psychosis it was observed that highly unsaturated fatty acids levels are reduced in the erythrocyte membranes and in the post-mortem brain tissue in comparison to healthy controls [12,13,14,15]. A decrease of the proportion of omega-3 fatty acids in the cellular membranes was associated with worse functioning also prior to the onset of psychosis [15] and could be linked with poor therapeutic response and greater severity of symptoms in patients with schizophrenia [16,17]. Schizophrenia is a psychotic disorder defined by abnormalities in five symptom domains: Delusions, hallucinations, disorganized thinking, disorganized behaviors and negative symptoms. The disturbance must be present continuously for more than six months and has usually a chronic course.

In order to evaluate the effects of omega-3 fatty acids in the different phases of schizophrenia, investigations were conducted in subjects at high risk to develop psychosis, patients with first psychotic episode and patients in chronic phase.

Five publications concerning the efficacy of omega-3 fatty acids in high-risk subjects for psychosis are available [18,19,20,21,22]. Four of these presented data from the same sample [18,19,20,21] and supported the utility of EPA and DHA (doses ranged between 1.2 g/day and 2.2 g/day) compared with the placebo in reducing the rate of progression in psychosis in short-term (12 weeks) and long-term (mean 6.7 years) studies. On the other hand, a multicentre double-blind randomized study [22] comparing omega-3 fatty acids plus cognitive-behavioral case management (CBCM) or placebo plus CBCM in a wide sample of young subjects at risk for developing psychosis reported an improvement in transition rates to psychosis with no significant differences between the two groups. A possible explanation could be that amelioration is due to the effect of CBCM and antidepressants that both groups received and that could have hidden the effects of omega-3 fatty acids. Results are summarized in Table 1.

In the first episode of psychosis (FEP), seven publications with data from three clinical studies have examined the effects of omega-3 fatty acids [23,24,25,26,27,28,29]. Trials performed by Berger and colleagues [23] and Pawelczyk and colleagues [27] tested EPA and DHA in adjunction to antipsychotics, while Emsley’s trial administered fatty acids as single therapy [26]. All studies used pure EPA or a fatty acids composition with high EPA. Duration of these trials ranged between 12 and 26 weeks. PUFAs dosages ranged between 2 and 3 g/day. In the study performed by Emsley et al. omega-3 fatty acids were administered to prevent relapse during antipsychotic discontinuation in remitted FEP followed for 2 years.

Reports from the two studies by Berger and Pawelczyk [23,24,25,27,28,29] reported encouraging data on EPA and DHA in first episode of psychosis in terms of symptoms reductions and neurobiological changes. Findings showed an improvement of general, psychotic, negative and depressive symptoms [24,25,27,28], a reduction of deterioration of hippocampus tissues with a positive effect on negative symptoms [25], a decrease of oxidative stress status of plasma with a positive effect on global and negative symptoms [28] and an increase of telomerase levels in peripheral blood cells with a positive effect on depressive symptoms and severity of illness [29]. RCT performed by Berger et al., 2008 was extended until 24 weeks of omega-3 fatty acids supplementation [24] and reported significant improvements in both symptoms and real-world functioning. Emsley’s study [26] did not show significant benefit for symptoms and functioning or relapse rate after antipsychotic discontinuation. This study was rather different from other RCTs as tested the efficacy of fatty acids in monotherapy (with no antipsychotic support) in relapses prevention. Results are summarized in Table 1.

Nine RCTs were performed in patients with stable schizophrenia [30,31,32,33,34,35,36,37,38]. Seven studies showed a positive effect of omega-3 fatty acids on several symptom domains of schizophrenia [30,31,32,34,36,37,38], while two trials [33,35] failed to observe significant advantages with PUFAs supplementation. The majority of studies tested the efficacy of EPA in adjunction to antipsychotics and only three studies [30,37,38] evaluated the effects of combination of EPA and DHA as adjuvant antipsychotic treatment. Duration of studies ranged between 8 and 16 weeks. Daily doses of PUFAs were from 0.9 to 4 g. EPA has been found superior than the placebo and also than DHA in reducing positive symptoms [30,31,32,34], negative symptoms [32], depressive symptoms [31,38] and anxious symptoms [38] of schizophrenia. One study indicated that omega-3 fatty acids were useful in reducing violent behaviors in schizophrenia [37]. Moreover, supplementation with EPA showed a significant decrease of impairment of the course of psychosis [36].

In spite of these encouraging data, it is hard to draw any conclusions on medium- and long- term efficacy of omega-3 fatty acids in stable schizophrenia. In fact, trials performed in this phase of illness have too short a duration to establish long lasting effects of these agents. It is interesting to notice that the majority of studies found a positive effect of pure EPA or a fatty acids composition with predominant EPA, at least when added to a stable antipsychotic treatment. On the contrary, the study by Peet and colleagues [30] tested pure DHA without finding significant effects.

Several reviews and meta-analyses [39,40,41,42,43,44,45] of RCTs concerning the efficacy of omega-3 in psychosis concluded that the evidence in favor of omega-3 fatty acids as psychotropic agents in schizophrenia is preliminary and findings remain inconclusive as available trials suffer from some significant limitations, including rather small sample size, heterogeneity in diagnosis and omega-3 combinations and doses and short duration of the majority of studies. Collected data suggested that EPA or a composition of fatty acids with high EPA could be more effective in the early phases of schizophrenia than in chronic phases of the disorder. Studies using pure or predominant EPA in high-risk subjects are lacking and should be performed. Results are summarized in Table 1.

### 4.2. Omega-3 Fatty Acids in Major Depressive Disorder

Some investigations reported that patients with major depressive disorder (MDD) have a lower level of EPA and DHA in their peripheral tissues (plasma, serum and red blood cells) than control subjects [46,47]. A dietary intake of fish or PUFAs could be linked to a decreased risk of major depressive disorder (MDD) [48,49,50,51,52,53] and improvement in white matter integrity [54]. Moreover, the anti-inflammatory capacity of omega-3 fatty acids, particularly EPA, could be crucial to prevent depression development [55]. Major depressive disorder is an episodic, recurrent disorder characterized by evident depression of mood and significant impairment of cognitive abilities and neuro-vegetative functions that last more than two weeks, but usually several months. Remission occurs between two episodes.

Thirty-six studies were published in order to evaluate the efficacy of EPA and DHA in mild and moderate unipolar depression [56,57,58,59,60,61,62,63,64,65,66,67,68,69,70,71,72,73,74,75,76,77,78,79,80,81,82,83,84,85,86,87,88,89,90,91]. Among these, eight were performed in women with perinatal depression [58,64,66,68,71,81,82,88]. Three studies were conducted in adolescents [62,86,87] and three trials in elderly depressive patients [72,73,75]. To limit the heterogeneity of this review, we decided to exclude trials on the efficacy of PUFAs in treating MDD in subjects with medical comorbidity and trials in which depressive symptoms were secondary to dementia and other neurological diseases. Fatty acids were administered both as monotherapy and as supplementation to ongoing pharmacotherapy or psychotherapy. The majority of studies tested the efficacy of combination of EPA and DHA. Three trials evaluated the efficacy of single EPA [56,57,70], one study compared single EPA and combination of EPA and antidepressants [65], two trials compared single DHA and placebo [58,59]. Duration of RCTs ranged between 4 and 16 weeks. Doses ranged from 0.4 to 4.4 g/day of EPA and from 0.2 to 2.4 g/day of DHA. To assess the level of depressive and depression-related symptoms rather heterogeneous evaluation instruments were used (Hamilton Depression Rating Scale, Montgomery-Asberg Depression Rating Scale, Beck Depression Inventory, Childhood Depression Rating Scale, Childhood Depression Inventory, Edinburgh Postnatal Depression Scale, Geriatric Depression Scale and Hopkins Symptom Checklist Depression Scale).

Concerning perinatal depression, available evidence consists of a prevalence of negative findings. RCTs performed in healthy pregnant women [68,71] or in pregnant women with major depressive disorder [64,66] did not find any significant effects on depression scores after childbirth. In a similar way, no significant therapeutic effect was observed in women with a history of post-partum depression treated from pregnancy to postpartum (up to 6 months) or after childbirth with EPA and DHA [58,59,81]. It should be noticed that two of these studies investigated single DHA and DHA has been found ineffective in treating depressive symptoms. Only two RCTs reported that combination of EPA and DHA supplementation during pregnancy could lower depression scores after childbirth [82,88].

In adolescence, two studies reported the efficacy of combination of EPA and DHA in reducing depressive symptoms [62] and behavioral symptoms, such as hyperactivity, impulsivity and opposition that often occur with depression in youths [86]. On the contrary one RCT [87] concluded that in adolescents with major depression omega-3 fatty acids did not appear superior to the placebo.

In elderly age, two studies of patients with mild to moderate depression showed an improvement of mood after treatment with low doses of both EPA and DHA [72,73,75].

Across other RCTs (21) that have been conducted in unipolar depressed adult patients (age ranged between 20 and 65 years), fifteen [56,57,60,65,70,74,77,78,79,80,83,85,89,90,91] trials found that EPA and DHA were efficacious in reducing depressive symptoms, while seven [59,61,63,67,69,76,84] studies obtained negative results. Studies that evaluated specifically the efficacy of EPA versus placebo [70] and EPA plus DHA versus placebo [85] showed a reduction of depression in patients treated with both PUFAs, but results did not reach statistical significance.

Two studies reported a significant improvement of depressive symptoms in patients undergoing antidepressant therapy treated with supplementation of single EPA at doses ≤ 2 g/day [56,57]. Only one study on suplementation of single DHA failed to show a significant effect of this agent on mood modulation [59].

The majority of trials compared the combination of EPA and DHA in addition to standard antidepressant therapy or in monotherapy versus placebo. Two studies [61,63] obtained no significant improvement in symptoms of depression in patients who were treated with similar doses of EPA and DHA (ranging from 2 to 3 g/day) and two studies [67,76] confirmed inefficacy of these agents on both depression and cognitive functions. In other investigations, omega-3 fatty acids supplementation was found superior to the placebo in ameliorating not only depressive symptoms [74], but also anxiety, sleep and regulation of emotions [89]. In particular, a combination of higher dose of EPA with lower dose of DHA revealed a beneficial effect on mood [72,79], while the opposite combination (lower EPA and higher DHA) failed to improve symptoms [60,61,63,67,83,85]. Studies that investigated the efficacy of EPA compared with DHA reported mixed results. In fact, one trial showed greater efficacy of EPA over DHA and over the placebo as an adjunctive treatment in mild and moderate depression [80], while another study failed to demonstrate a superior antidepressive effect of either omega-3 fatty acids (DHA and EPA) over the placebo [84]. One study [91] demonstrated that subjects with major depression and a high number of inflammatory biomarkers had a better response to EPA than the placebo and a lower response to DHA than the placebo. Other RCTs evaluated the effect of EPA or EPA plus DHA in monotherapy or in association with antidepressants drugs (fluoxetine and citalopram). Results suggested that the efficacy of EPA and fluoxetine combination was superior to either of them alone (fluoxetine or EPA) in reducing depressive symptoms [65]. The add-on therapy to citalopram with EPA, DHA and other PUFAs was found significantly more efficacious in reducing depression than single therapies, but with no changes in rapidity of initial antidepressant response [77].

The dedicated section “Complementary and Alternative Medicine Treatment” of the Canadian Network for Mood and Anxiety Treatments (CANMAT) clinical guidelines for the management of adult with major depressive disorder included the results of four meta-analyses [92,93,94,95] and two systematic reviews [96,97]. One meta-analysis [92] did not find any benefit from omega-3 fatty acids treatment; one meta-analysis [94] and one review [97] reported discordant and equivocal outcomes; one meta-analysis [95] stated positive outcomes for EPA and DHA as monotherapy; and one meta-analysis [93] and one review [96] found a positive outcome of PUFAs as adjunctive therapy. The first meta-analysis by Bloch and Hannestad [92] presented negative conclusions, but this result was likely influenced by the inclusion of a study in which strict criteria for depression were not met [67]. If this study is not considered, the meta-analysis has positive results.

On the basis of these findings, CANMAT guidelines concluded that PUFAs reached level 1 evidence (meta-analysis with narrow confidence intervals and/or two or more RCTs with adequate sample size, preferably placebo controlled) of efficacy but, because of the inconsistency in the findings, are recommended as second-line monotherapy for mild to moderate MDD and as adjunctive therapy to antidepressants for moderate to severe MDD [98]. After publication of the CANMAT guidelines, other five systematic reviews were published [99,100,101,102,103]. Authors concluded that quality of evidence was not so good to determine the effectiveness on PUFAs in treatment of MMD. However, there is a general agreement to consider omega-3 PUFAs as a promising supplementation therapy in combination with antidepressants or in monotherapy. A focal point to be considered is the different efficacy of the two fatty acids more commonly tested, EPA and DHA. Two meta-analyses published by Martins [104] and Sublette and colleagues [105] reported that EPA is likely more effective than DHA in treating depression and that combination of the two fatty acids has antidepressant effects when the proportion of EPA is more than 60%. So, available evidence indicates that pure or predominant EPA, but not pure DHA, is effective in major depression.

In conclusion, supplementation with PUFAs in patients with major depression seems useful in improving depressive symptoms, but findings are not univocal. Data in adolescents and elderly patients are promising, but more studies investigating sufficiently large samples are needed. On the contrary, EPA and DHA failed to show significant effects on depressive symptoms in perinatal depression. Data of studies of major depression are summarized in Table 2.

### 4.3. Omega-3 Fatty Acids in Bipolar Disorder

A growing number of investigations reported that inflammatory mechanisms are important mediator of pathophysiology in bipolar disorder [7,106]. Omega-3/omega-6 PUFAs ratio, implicated in processes of inflammation, is often unbalanced in patients with affective disorders. In particular, in subjects with a diagnosis of bipolar disorder lower erythrocyte membrane levels of omega-3 fatty acids have been observed [107,108]. Bipolar disorder is the modern definition of the classic concept of manic-depressive psychosis. It is characterized by manic or hypomanic episodes with an abnormal state of euphoria or excitement and in most cases by alternating major depressive episodes. Free intervals between episodes are often present and last for varying periods.

Five RCTs were conducted in order to verify the effect of supplementation with EPA, DHA and their combination in bipolar disorder in addition to mood stabilizing and antipsychotic therapies [109,110,111,112]. One RCT tested EPA plus DHA as monotherapy versus placebo [113] and one study evaluated the efficacy of alfa linolenic acid (ALA) in addition to other psychotropic medications in children and adolescents with bipolar disorder [114]. Trials duration ranged between 4 and 16 weeks. Daily doses ranged from 0.4 to 6.2 g/day of EPA and from 0.2 to 3.4 g/day of DHA. In the study performed by Gracious et al. ALA was administered at the dose of 0.5 g/day. Evaluation instruments were rather homogeneous across studies (Hamilton Depression Rating Scale and Young Mania Rating Scale).

Among these seven RCTs, only two studies showed a significant effect of EPA and DHA on depressive symptoms in bipolar disorder [111,113]. Patients treated with a combination of EPA and DHA had a significantly longer period of remission than those treated with the placebo, with significant improvement and remission of depressive symptoms [113] EPA in addition to standard pharmacotherapy resulted superior to the placebo in reducing depressive symptoms in bipolar depression [111]. Other five studies failed to show significant differences between single EPA, single ALA or EPA plus DHA versus placebo on manic or depressive symptoms [107,109,110,113,114].

Four systematic reviews [7,105,115,116] and two meta-analyses [117,118] concluded that scarcity of studies, rather small sample size and heterogeneity of trials duration and PUFAs dose represented important limitations that did not allow to obtain reliable data on this topic. In summary, results provided only initial evidence that omega-3 fatty acids in addition to standard medications could be useful on depressive, but not manic symptoms of bipolar disorder [10]. Data of studies of bipolar disorder are summarized in Table 3.

### 4.4. Omega-3 Fatty Acids in Anxiety Disorders

Similarly to major depression and bipolar disorder, inflammatory response is pertinent to the pathophysiology of anxiety. As anxiety is linked to augmented production of pro-inflammatory cytokines and low levels of PUFAs in blood, omega-3 supplementation could induce a reduction of anxious symptoms by decreasing inflammatory processes [119,120,121]. Anxiety disorders are a group of disorders that shared symptoms of excessive or unreasonable fear and anxiety and related behavioral abnormalities and neuro-vegetative symptoms.

RCTs available in literature on anxiety were performed in subjects with anxious symptoms in other psychiatric disorders (ADHD, borderline personality disorder, major depressive disorder, premenstrual syndrome, Tourette syndrome, substance abuse, Parkinson and Alzheimer diseases). To our knowledge, there are not RCTs that have systematically investigated the effect of omega-3 PUFAs in anxiety disorders. Only a small trial [122] found a significant effect of EPA and DHA (doses of EPA 2.2g/day and DHA 0.5g/day) on anxiety and inner tension in a sample of substance abusers. The trial lasted 12 weeks. Anxiety continued to significantly decrease in the active treatment group after three months discontinuation.

On this topic, one review [120] and one meta-analysis [123] were published. The review was focused on the possible mechanisms of action of omega-3 PUFAs on anxiety. Results reported that omega-3 fatty acids influence anxious symptoms by interleukin-6, neuroendocrine protein BDNF and cortisol modulation [120]. The meta-analysis highlighted a modest anxiolytic effect of omega-3 fatty acids in patients with several neuropsychiatric disorders or major physical illnesses [123].

### 4.5. Omega-3 Fatty Acids in Obsessive-Compulsive Disorder

Obsessive-compulsive disorder is characterized by the presence of recurrent and intrusive thoughts (obsessions) and repetitive behaviors (compulsions) that patients are compelled to act in response to obsessions.

No recent RCTs were published on supplementation of omega-3 fatty acids in obsessive-compulsive disorder. The only available study was included in our previous review [10]. Results were unfavorable as EPA (2 g/day) in augmentation of a stable dose of SSRIs (paroxetine or fluvoxamine or fluoxetine) for 6 weeks was not associated to significant improvement of anxious, obsessive-compulsive and depressive symptoms compared with the placebo [124]. Data are summarized in Table 4.

### 4.6. Omega-3 Fatty Acids in Post-Traumatic STRESS Disorder (PTSD)

Several investigations showed that PTSD is associated to a persistent and increased oxidative stress with accelerated cellular aging. Loss of neural integrity in the hippocampus, amygdala, medial prefrontal and anterior cingulate cortex and lower levels of PUFAs, in particular DHA, were found in patients with PTSD [129,130]. A possible mechanism of action of these psychotropic agents in PTSD symptoms is that they can facilitate fear-extinction learning and memories by facilitating hippocampal neurogenesis [131,132,133,134]. In addition, PUFAs could reduce sympathetic nerve activity implicated in the development of PTSD [135,136,137,138]. Post-traumatic stress disorder is defined by the onset of varying symptoms following exposure to traumatic experiences. Re-experiencing emotional disturbances and behavioral symptoms dominate clinical picture. While in recent years the interest for both PUFAs efficacy and PTSD treatment has largely increased, RCTs that have evaluated the therapeutic role of EPA and DHA in subjects with PTSD are still scarce. To our knowledge, only three studies are available [125,126,127]. Two of these studies conducted two different analyses on patients of the same trials [125,126]. Results on the efficacy of DHA are controversial. The first trial showed that DHA supplementation was not superior to the placebo in preventing PTSD symptoms at 12 weeks after traumatic event [125], but the re-analysis demonstrated that symptoms were improved in parallel with an increase in blood EPA levels [126]. The second trial found that DHA at high dose (1.4 g/day) with addition of low dose of EPA (0.1 g/day) improved tachycardia, but not psychiatric symptoms of PTSD [127].

Due to the scarcity of data we cannot draw any conclusion on the opportunity to use omega-3 fatty acids in PTSD and further investigations are required collect data on this issue. In particular, some findings suggest to test a composition of PUFAs with dominant EPA. Data of studies of PTSD are summarized in Table 4.

### 4.7. Omega-3 Fatty Acids in Substance Use Disorder

Some investigations reported that inflammatory cascade induced by cytokines can be responsible for the physical and psychological symptoms concomitant to craving. It is possible that omega-3 fatty acids, such as EPA, neutralize these toxic effects in the brain [122,139]. To our knowledge, no recent studies have been published after our previous review [10] on the efficacy of omega-3 fatty acids in the treatment of substance use disorders. The only two studies available were conducted by the same research group and showed that combination of EPA and DHA at high dose (3 g/day) produced a positive effect on anxious symptoms related to addiction in 12 weeks of supplementation and during a follow-up period of further 12 weeks [122,128].

Data in this field are too scarce to provide any reliable indication on the efficacy of PUFAs in patients with substance use. Data of studies of substance use disorder are summarized in Table 4.

### 4.8. Omega-3 Fatty Acids in Attention Deficit Hyperactivity Disorder (ADHD)

Recent investigations suggested a significant role of PUFAs on the immune system that is involved in ADHD. PUFAs, in particular DHA deficiency in rats is involved with a change in behavioral manifestations, such as increased motor activity and decreased learning ability [140,141]. In humans, omega-3 fatty acids deficit in fetal brain could impinge normal development and represent a modifiable risk factor for ADHD [142]. Attention deficit hyperactivity disorder is defined by a persistent condition of lack of attention and hyperactivity or impulsivity that compromises functioning or development.

Fourteen RCTs were published on this topic [143,144,145,146,147,148,149,150,151,152,153,154,155,156]. Eleven studies tested the association of EPA and DHA versus placebo with no other ADHD medications (stimulant or not stimulant drugs) [144,145,146,147,148,149,150,153,154,155,156]. One trial evaluated the effect of DHA versus placebo in patients who received other ADHD medications [143], one study tested EPA versus placebo in patients receiving ADHD medications [152] and one RCT studied the efficacy of EPA versus placebo in subjects receiving no other medications [151].

EPA and DHA were administered at a daily dose ranging between 0.08 and 0.65 g for EPA and 0.04 and 0.64 g for DHA. Duration of trials ranged between 12 and 30 weeks. Evaluation instruments for patients’ assessment were homogeneous enough among studies. Most common rating scales were: Conners’ parenting rating scale and Conners’ teacher rating scale; disruptive behavior disorders; conduct for parents and the disruptive behavior disorders; attention for teachers. Parental and teachers’ ratings of symptoms showed weak consistency. A hypothesis to explain this discordance could be that parents are more likely to observe changes in the child’s routine (i.e., daily activities such as getting ready for school, getting dressed, completing homework), while teachers provided a report of the children’s behavior at school, in terms of peer interactions and relationships. So, settings and child’s activities that were evaluated by parents and teachers were significantly different.

Five studies did not find significant differences between EPA and DHA versus placebo in improving ADHD symptoms in terms of hyperactivity and cognitive performances [143,145,146,149,154]. Several other trials reported that PUFAs supplementation significantly improved parental reports of global ADHD symptoms [144,147,148,151,152,155,156], inattention [144,147,151,152,153,155,156] and hyperactivity [151,152,155]. In particular, higher doses of EPA (superior to 0.5 g/day) were found effective on hyperactivity [151,152,155] and in one study single EPA was shown efficacious on both hyperactivity and inattention [152]. On the other hand, omega-3 fatty acids did not reach a significant effect in reducing teacher’s measures of inattention, hyperactivity and ADHD global symptoms [151,153,155].

Concerning cognitive performances, omega-3 fatty acids supplementation showed efficacy in improving omission and commission errors in cognitive tasks [143,147,148], but not memory and information processing [143,147,148,155].

Two meta-analyses [157,158] concluded that omega-3 fatty acids supplementation in ADHD produced small to modest significant reductions of symptoms, in particular when high doses of EPA were used [157]. Among the three reviews available on this topic, the first was published by Gillies and colleagues [159] and found little evidence that PUFAs had positive effects on the symptoms of ADHD in children and adolescents. There was only limited indication that a combination of omega-3 and omega-6 fatty acids induced overall improvement. Two more recent reviews performed by Gow et al. [160] and Chang et al. [161] reported more favorable conclusions, as they found some evidence of efficacy of omega-3 fatty acids on symptoms and cognitive performances of ADHD patients. The different conclusions of these reviews are difficult to explain, as no new trials were conducted in recent years. Maybe differences in studies selection and data analysis are responsible for these discrepancies. Data of studies of ADHD are summarized in Table 5.

### 4.9. Omega-3 Fatty Acids in Autism Spectrum Disorders

As some evidence suggested that autism may involve a cellular functional deficiency or imbalance of omega-3 fatty acids [162,163,164], the number of investigations on the role of PUFAs as a treatment option for autism spectrum disorders has grown up in the last years. Autism spectrum disorders are characterized by prolonged deficits in social communication and relationships and by restricted repetitive patterns of behavior and activities.

Eight RCTs are available [165,166,167,168,169,170,171,172]. Four of these trials tested the efficacy of combination of EPA and DHA versus placebo at doses between 0.5 and 0.8 g/day for EPA and 0.2 and 0.7 g/day for DHA [165,166,167,170], one study evaluated single DHA versus placebo [169], one compared a large dose of arachidonic acid plus DHA or the placebo [168], one tested omega-3 fatty acids (EPA and DHA) and omega-6 fatty acids (gamma linolenic acid) versus placebo [171] and one evaluated the efficacy of vitamin D, DHA and both agents versus placebo [172]. Duration of trials varied from 6 to 24 weeks. Five trials [165,166,167,168] assessed symptoms with the Aberrant Behavior Checklist, three [166,169,170,172] used the Behavior Assessment System for Children and four [167,168,172] used the Social Responsiveness Scale.

Results showed that omega-3 fatty acids supplementation improved some core symptoms of autism, in particular hyperactivity [165,166,167] lethargy [165,166,168] and stereotypy [165,166,167,168]. One trial reported a significant improvement in symptoms of irritability and in social domains (social awareness and social communicative functioning) [172]. A possible beneficial effect of the association of omega-3 and omega-6 fatty acids was found in language development in one study performed in preterm children exhibiting autism spectrum disorder symptoms [171].

The two available meta-analyses on efficacy of PUFAs in autism spectrum disorders are discordant in their conclusions. The first [173] stated that no significant differences in severity of autism symptoms were observed after treatment with PUFAs, except for lethargy in some studies. The second meta-analysis [174] suggested a small but positive effect of omega-3 fatty acids in reducing hyperactivity. Data concerning lethargy and stereotypy are statistically weak and need further confirmations.

In conclusion, the role of PUFAs in treatment of autism spectrum disorders is still debated. The most favorable data concern the reduction of hyperactivity, followed by those supporting the improvement of lethargy. Data of studies of autism spectrum disorders are summarized in Table 6.

### 4.10. Omega-3 Fatty Acids in Anorexia Nervosa

Abnormal levels of omega-3 and omega-6 fatty acids were observed in patients with anorexia nervosa and in the activity of the enzyme responsible for desaturation of fatty acids [175,176]. Anorexia nervosa is defined by three fundamental disturbances: Pronounced decreased of food intake, fear of increase in weight and alteration in self-perception of shape. Several studies evaluated whether the peripheral profile of PUFAs was different in subjects with eating disorders and healthy controls [177,178,179,180]. Five RCTs were performed to assess the efficacy of omega-3 in treating anorexia nervosa [181,182,183,184,185]. In all studies patients were predominantly constituted by females (85–100%). A wide range of omega-3, omega-6 and omega-9 fatty acids was administered in the trials (alpha-linolenic acid, EPA, DHA, stearidonic acid, adrenic acid, arachidonic acid, gamma linolenic acid, oleic acid). All articles used the Eating Disorders Inventory to measure eating symptoms. Duration of trials ranged between 8 and 12 weeks. Daily dose ranged between 0.3 and 2.1g of EPA and between 0.2 and 0.6 g of DHA. Is it hard to ascertain the daily dosages of other mixed PUFAs (omega-6, omega-9 fatty acids) used in the trials.

For patients with this diagnostic category findings were globally unfavorable, as all RCTs did not find significant changes before and after PUFAs supplementation of eating symptoms, mood and anxiety. Two studies [181,183] registered a significant change in body weight of patients with anorexia after omega-3 fatty acids supplementation.

One recent meta-analysis was performed with a twofold objective: (1) To compare the differences of PUFAs levels in patients with eating disorders and healthy subjects; (2) to verify potential beneficial effects of these agents on eating dysfunction and related symptoms [186]. In conclusion, authors found that there was an abnormal level of PUFAs in peripheral blood and tissues in patients with eating disorders. In particular, PUFAs levels were higher in patients than in controls, except for lower levels of omega-6 fatty acids. Concerning clinical effects, omega-3 fatty acids were active only in improving body weight in anorexia, but no significant changes were observed in severity of eating dysfunction or mood symptoms related to eating disorders. So, there is probably no point in specific supplementation of omega-3 fatty acids. Data of studies of anorexia nervosa are summarized in Table 7.

### 4.11. Omega-3 Fatty Acids in Borderline Personality Disorder

Among personality disorders, the effect of omega-3 fatty acids was tested only in borderline personality disorder. Borderline personality disorder can be defined a pervasive and long-lasting condition of dysfunctional relationships, distorted self-image and unstable affective states and impulsive-behavioral dyscontrol. For several years the nosological distinction between this disorder and bipolar disorder was debated in research community. While there are some overlapping symptoms including emotional instability and impulsivity, recent studies suggested that borderline personality disorder and bipolar disorder can be distinguished as separate clinical entities [187]. As several investigations showed a positive effect of omega-3 fatty acids on symptoms of impulsivity and aggression in healthy and psychiatric subjects [188,189,190], efficacy of supplementation of these agents has been verified in patients with borderline personality disorder, who often show impulsive-behavioral dyscontrol and aggressiveness. To our knowledge five RCTs have been published [191,192,193,194,195]. Trials were rather heterogeneous in choice of diagnostic criteria and contemporary administration of conventional medications.

One study tested the efficacy of single EPA [191] while the remaining evaluated the effects of combination of EPA and DHA. Daily doses ranged from 1 to 1.2 g for EPA and from 0.8 to 0.9 for DHA. RCTs duration ranged from 8 and 12 weeks [191,192,193]. Two studies were follow-up studies: One lasted 10 years [194] and one lasted 6 months [195]. Evaluation instrument to assess aggressiveness is homogeneous (Modified Overt Aggression Scale (MOAS), while other rating scales are rather different across trials (Montgomery-Åsberg Depression Rating Scale, Beck Depression Inventory, Borderline Personality Disorder Severity Index, Barratt Impulsiveness Scale).

Omega-3 fatty acids with no adjunctive medications were found efficacious on aggression and depressive symptoms [191] in comparison with the placebo. Moreover, these agents were shown effective in addition to standard pharmacotherapies in reducing depressive symptoms, self-injuries [192,193,194], impulsivity [192,193,194] and outbursts of anger [193,195].

No reviews or meta-analyses were performed specifically on the use of omega-3 fatty acids in personality disorders. Nevertheless, the Cochrane systematic review for the pharmacological treatment of borderline personality disorder [196,197] concluded that omega-3 fatty acids (EPA and DHA) were a therapeutic option for this personality disorder that had obtained some evidence of efficacy, together with antipsychotics and mood stabilizers. Data of studies of borderline personality disorder are summarized in Table 8.

## 5. Adverse Effects

About 80% of RCTs included in this review evaluated adverse effects of PUFAs. Tolerability was generally good for omega-3 fatty acids among studies. In clinical trials patients reported mild nausea, diarrhea and a fishy aftertaste as main adverse events, but they did not induce discontinuation [74,198,199]. These agents were recognized as safe also by the Panel of The European Food Safety Authority (EFSA) that stated that EPA and DHA supplementation up to 5 g/day for a maximum of 16 weeks is not dangerous for the general population [197], in terms of cardiovascular and bleeding risk [200,201,202], glucose regulation [203,204,205], risk of activation of inflammatory response [206], lipid peroxidation and cardiovascular risk [207]. However, patients on anticoagulant and antiplatelet medications require additional monitoring during PUFAs supplementation [208].

Among RCTs included in this review there were no reports of severe and impairing adverse events with EPA and DHA. In general, side effects registered in these trials were related to concomitant psychotropic medications. High retention and adherence was reported (>80%).

## 6. Conclusions

The present article is an update of our previous review [10] and is aimed to investigate the use of PUFAs in treatment of psychiatric disorders. Our search was focused on randomized control trials that have been conducted in this field over the last four decades.

While the role of PUFAs, in particular EPA and DHA, in psychiatric disorders has received in recent years a growing interest and has been investigated in an increasing number of clinical trials, a general agreement about their efficacy is lacking and the available evidence is controversial and mainly inconclusive. One of the main obstacle to draw more definitive conclusions about the effects of these agents consists of the wide heterogeneity across RCTs. Differences in methods are conspicuous and concern sample size, diagnostic criteria, type and doses of PUFAs (i.e., EPA or DHA or combination of the two omega-3 fatty acids or addition of omega-6 or omega-9 fatty acids), association with standard medications, trials duration and follow-up evaluations.

The main evidence of efficacy for EPA and DHA has been obtained in mood disorders. In particular, supplementation with omega-3 fatty acids was found efficacious in reducing depressive symptoms in major depressive disorders and, in association with standard medications, in bipolar disorder. Initial data in adolescents and elderly depressed patients are promising, but further studies in larger samples are required. On the other hand, EPA and DHA failed to improve depressive symptoms in perinatal depression. It is noticeable that the Canadian Network for Mood and Anxiety Treatments (CANMAT) clinical guidelines for the management of adults with major depressive disorder attributed to PUFAs level 1 evidence of efficacy in depression, but precautionary recommended these agents as second-line monotherapy for mild to moderate major depression and as adjunctive therapy to antidepressants for moderate to severe major depressive disorder.

Results concerning schizophrenia and related psychotic disorders are still debated and available evidence does not allow either to reject or support the use of omega-3 fatty acids in psychotic patients. Omega-3 fatty acids supplementation could be more efficacious in the early phases of schizophrenia (first episode of psychosis) than in chronic phases of the disorder in addition to antipsychotic treatment. In particular, EPA and DHA could be useful in reducing the progression of illness in subjects at high risk to develop psychosis.

In borderline personality disorder supplementation of omega-3 fatty acids obtained favorable results on some core symptoms: Impulsivity, self-injuries and anger. Trials in this field are promising, but are still limited. Small to modest beneficial effects of omega-3 fatty acids in ADHD were found by several studies that were performed in this disorder. In particular, studies that tested EPA at high doses or the association of omega-3 and omega-6 fatty acids got more favorable findings in terms of reduction of hyperactivity and, to a lesser degree, improvement of cognitive performance. In autism spectrum disorders the number of clinical trials and reviews increased since our previous review [10], but a consensus among investigators has not been obtained, yet. Most favorable findings concern the reduction of hyperactivity, followed by the improvement of lethargy in children with autism. Omega-3 supplementation cannot be recommended as an alternative to support behavioral therapies for autism spectrum disorder. It can be proposed only to complement other therapies in this clinical population. Efficacy of omega-3 fatty acids was not specifically assessed in patients with anxiety disorders (i.e., panic disorder, social phobia, agoraphobia), but a modest anxiolytic effects of omega-3 fatty acids in patients with several neuropsychiatric disorders or major physical illnesses was observed. PTSD is a disturbance that has acquired considerable interest in the last decades, but studies on the use of PUFAs in these patients are too scarce to propose any indication. PUFAs were not found efficacious in treating symptoms of obsessive-compulsive disorder, eating disorders and substance use disorder.

Concerning tolerability, the large majority of RCTs reported that omega-3 fatty acids were well tolerated. The most common, but mild side effects reported in clinical trials were mild nausea, diarrhea and a fishy aftertaste. The intake of EPA and DHA up to 5 g/day and up to 16 weeks seems to be safe in terms of bleeding risk, glucose regulation, infection risk and lipid alterations.

While the results are not consistent enough, available findings on the effects of omega-3 fatty acids in several psychiatric disorders are promising in terms of clinical efficacy and good tolerability. In our opinion, a fundamental issue remains unsolved. When (in what kind of psychiatric disorder? at what level of severity of symptoms?) can these agents be administered in monotherapy and in which cases is the association with standard pharmacotherapies and/or psychosocial interventions preferable? In addition, which medications and which models of psychotherapy are better indicated in combination with PUFAs to treat specific psychiatric disorders? To date, these questions cannot be answered as the majority of trials do not provide specific information on the adjunctive therapies (doses, duration, type of medications or psychotherapies).

In conclusion, a major challenge for researchers in this field is to design and conduct further RCTs with rigorous methods to definitively demonstrate that omega-3 fatty acids supplementation can be considered a reliable option to treat some specific psychiatric disorders and to make clear which are the better modalities to provide these agents in single clinical conditions.

## Figures and Tables

**Table 1:** Double-blind controlled trials of polyunsaturated fatty acids (PUFAs) as add-on strategy in the treatment of schizophrenia.

High-Risk Psychosis				
Study	Drugs and Dose	Sample	Treatment Duration	Results
Amminger et al., 2010 [18]	EPA 700 mg/day + DHA 480 mg/day	81 individuals UHR	12 weeks	↓ progression in psychosis in young UHR patients
Amminger et al., 2013 [19]	EPA 700 mg/day + DHA 480 mg/day + 7.6 mg vit. E	81 young individuals at UHR	12 weeks	↓ positive symptoms, negative symptoms and general symptoms, ↑ level of functioning
Amminger et al., 2015 [20]	EPA 700 mg/day + DHA 480 mg/day	81 young individuals at UHR	12 weeks	↓ both risk of progression to psychotic disorder and psychiatric morbidity
Smesny et al., 2014 [21]	EPA 700 mg/day + DHA 480 mg/day	81 young individuals at UHR	12 weeks	normalizing PLA2 activity and d-6-desaturase-mediated metabolism of o-3 and o-6 PUFAs
McGorry et al., 2017 [22]	EPA 840 mg/day + DHA 560 mg/day + CBCM	304 individuals UHR	24 weeks	not effective under conditions where evidence-based psychosocial treatment is available
**First-episode Psychosis**				
Study	Drugs and Dose	Sample	Treatment Duration	Results
Berger et al., 2007 [23]	Ethyl-EPA 3 g/day	69 patients	12 weeks	accelerated treatment response
Berger et al., 2008 [24]	EPA 2 g/day	24 patients	12 weeks	↓ of negative symptoms
Wood et al., 2010 [25]	EPA 2 g/day	17 patients	12 weeks	increased water in hippocampal tissues and positive effect on negative symptoms
Emsley et al., 2014 [26]	EPA 2 g/day + DHA 1 g/day + α-LA 300 mg/day	33 patients	2 years	relapse prevention of psychotic symptoms
Pawelzcyk et al., 2016 [27]	EPA + DHA 2.2 g/day	71 patients	26 weeks	↓ psychotic symptoms measured with PANSS ↓ depressive symptoms ↑ level of functioning
Pawelzcyk et al., 2017 [28]	EPA + DHA 2.2 g/day	71 patients	26 weeks	improved PANSS negative and general symptoms, along with global functioning
Pawelzcyk et al., 2018 [29]	EPA + DHA 2.2 g/day	71 patients	26 weeks	↑ level of telomerase in peripheral blood cells with ↓ depressive symptoms
**Stable Schizophrenia**				
Study	Drugs and Dose	Sample	Treatment Duration	Results
Peet et al., 2001 [30]	EPA or DHA 2 g/day	45 patients	12 weeks	↓ psychotic symptoms measured with PANSS in the group treated with EPA
Peet and Horrobin, 2002 [31]	E-EPA 1–4 g/day	115 patients	12 weeks	↓ positive symptoms measured with PANSS, ↓ depressive symptoms
Emsley et al., 2002 [32]	ethyl-EPA 3 g/day	40 patients	12 weeks	↓ positive symptoms and negative symptoms measured with PANSS
Emsley et al., 2006 [33]	ethyl-EPA 2 g/day	77 patients	12 weeks	no efficacy on specific psychotic symptoms
Jamilian et al., 2014 [34]	1 g/day	60 patients	8 weeks	↓ psychotic symptoms measured with PANSS
Fenton et al., 2001 [35]	ethyl-EPA 3 g/day	87 patients	16 weeks	no significant differences in positive, negative symptoms, mood or cognition
Bentsen et al., 2013 [36]	ethyl-EPA 2 g/day	99 patients	16 weeks	↓ impairment of the course of psychosis
Qiao et al., 2018 [37]	DHA 360 mg/day + EPA 540 mg/day	50 patients	12 weeks	↓ violence, but no improvement in positive and negative symptoms
Robinson et al., 2019 [38]	EPA 740 mg and DHA 400 mg/day	50 patients	16 weeks	↓ confusion, anxiety, depression, irritability and tiredness/fatigue

Abbreviations: EPA = eicosapentaenoic acid; DHA = docosahexaenoic acid; ethyl-EPA = ethyl-eicosapentaenoic acid; α-LA = α-lipoic acid; UHR = ultra-high risk; FEP = first episode of psychosis; ↓ = decrease of; ↑ = increase of.

**Table 2:** Double-blind controlled trials of polyunsaturated fatty acids (PUFAs) in the treatment of depressive disorders.

Study	Drugs and Dose	Sample	Treatment Duration	Results
Nemets et al., 2002 [56]	ethyl-EPA 2 g/day	20 patients	4 weeks	↓ depressive symptoms measured with HDRS from the second week of treatment
Peet and Horrobin, 2002 [57]	ethyl-EPA 1, 2 or 4 g/day add-on standard antidepressant treatment	70 patients resistant to antidepressant treatment	12 weeks	↓ depressive symptoms measured with HDRS, MADRS and BDI in the group treated with 1 g/day of PUFAs
LIorente et al., 2003 [58]	DHA 0.2 g/day monotherapy	99 healthy pregnant women	16 weeks	no effect on depression
Marangell et al., 2003 [59]	add on to standard therapy DHA 2 g/day monotherapy	36 depressed patients	12 weeks	no significant differences
Su et al., 2003 [60]	ethyl-EPA 4.4 g/day + DHA 2.2 g/day add-on existing antidepressant treatment	22 patients	8 weeks	↓ depressive symptoms measured with HDRS
Silvers et al., 2005 [61]	EPA 0.6 g/day +DHA 2.4 g/day added to standard therapy	77 MDD patients	12 weeks	no evidence that n-3PUFAs improved mood compared to placebo. Mood improved in both groups within the first 2 weeks of the study
Nemets et al., 2006 [62]	ethyl-EPA 0.4 g/day + DHA 0.2 g/day	20 patients 6–12 years-old	16 weeks	↓ depressive symptoms measured with CDRS, CDI and CGI
Greyner et al., 2007 [63]	EPA 0.6 g/day + DHA 2.2 g/day add to standard therapy	83 MDD patients	16 weeks	no significant differences
Freeman et al., 2008 [64]	EPA 1.1 g/day + DHA 0.8 g/day	59 women	8 weeks	no benefit on perinatal depressive symptoms
Jazayeri et al., 2008 [65]	EPA 1 g/day versus fluoxetine 20 mg/day	60 patients	8 weeks	↓ depressive symptoms in both groups
Rees et al., 2008 [66]	ethyl-EPA 0.4 g/day + DHA 1.6 g/day	26 pregnant patients	6 weeks	no benefits on depressive symptoms
Rogers et al., 2008 [67]	EPA 0.63 g/day + DHA 0.85 g/day monotherapy	218 mild to moderate depressed patients untreated	12 weeks	n-3PUFAs not have beneficial or harmful effects on mood in mild to moderate depression.
Doornbos et al., 2009 [68]	DHA 0.22 g/day or DHA 0.22 g/day + AA (0.22 g/day arachidonic acid) monotherapy	119 healthy pregnant women	28 weeks	red blood cell DHA, AA and DHA/AA ratio did not correlate with EPDS or blues scores
Lucas et al., 2009 [69]	EPA 1.05 g/day + DHA 0.25 g/day mono-therapy	120 patients with psychological distress with or without MDD in comorbidity	8 weeks	no significant differences
Mischoulon et al., 2009 [70]	EPA 1 g/day + (+0.2% dl alphatocopherol) monotherapy	57 MDD patients	8 weeks	↓ depressive symptoms assessed with HDRS, but no statistical significance
Makrides et al., 2010 [71]	DHA-rich tuna oil capsules 0.5 g/day mono therapy	2399 healthy pregnant women at 21 weeks’ gestation	women received assigned capsules daily, from study entry until birth of their child	DHA during pregnancy did not lower levels of postpartum depression
Rondanelli et al., 2010, 2011 [72,73]	EPA 1.67 g/day + DHA 0.83 g/day added to existing antidepressant treatment	46 elderly female residents in a nursing home	8 weeks	↓ depressive symptoms assessed with GDS, improvement of phospholipids fatty acids profile
Lespérance et al., 2011 [74]	EPA 1.05 g/day + DHA 0.15 g/day	432 patients with a major depressive episode	8 weeks	↓ depressive symptoms only for patients without comorbid anxiety disorders
Tajalizadekhoob et al., 2011 [75]	EPA 0.18 g/day + DHA 0.12 g/day add to standard therapy in 55 patients while in 11 monotherapy	66 patients with mild-to moderate depression aged > 66 years	24 weeks	low-dose n-3PUFAs have some efficacy in mild to moderate depression
Antypa et al., 2012 [76]	EPA 1.74 g/day+ DHA 0.25 g/day added to standard therapy	71 patients with history of at least one MDD	4 weeks	no significant effects on memory, attention, cognitive reactivity and depressive symptoms
Gertsik et al., 2012 [77]	EPA 0.9 g/day + DHA 0.2 g/day + other n-3 PUFAs (0.1 g/day) added to citalopram	42 MDD patients taking citalopram	9 weeks	significantly greater improvement in HDRS scores
Krawczyk et al., 2012 [78]	EPA 2.2 g/day + DHA 0.7 g/day + GLA (0.24 g/day) + vit. E added to standard therapy	21 patients with severe episode of treatment resistant recurring depression	8 weeks	n-3PUFAs significantly improved HDRS scores
Rizzo et al., 2012 [79]	EPA/DHA 2.1/2.5 g of n3-PUFA monotherapy	46 MMD patients (only women > 66 years old)	8 weeks	mean GDS score and AA/EPA ratio, in whole blood and RBC membrane phospholipids, were significantly lower
Mozzafarri Khoshari et al., 2013 [80]	EPA 1 g/day or DHA 1 g/day added to standard therapy	81 mild to moderate depressed patients	12 weeks	↓ HDRS score compared with those in the DHA or placebo groups
Mozurkewich et al., 2013 [81]	EPA 1.06 g/day+ DHA 0.27 g/day or EPA 0.18 g/day + DHA 0.9 g/day mono-therapy	126 healthy pregnant women	6-8 weeks	no differences between groups in BDI scores or other depression endpoints
Judge et al., 2014 [82]	DHA 0.3 g/day	42 healthy pregnant women	8 weeks	↓ depressive symptoms assessed with PDSS
Ginty et al., 2015 [83]	EPA + DHA 1.4 g/day monotherapy	23 depressed patients	3 weeks	n-3PUFAs group had a significant reduction in BDI scores over time
Mischoulon et al., 2015 [84]	EPA 1 g/day or DHA 1 g/day	196 patients	8 weeks	EPA and DHA were not superior to placebo
Park et al., 2015 [85]	EPA 1140 g/day + DHA 0.6 g/day add to standard therapy	35 MDD patients	12 weeks	no significant differences
Young et al., 2017 [86]	PEP + EPA 1.4g/day + DHA 0.2 g/day + 0.4 g/day other	72 patients 7–14 years old	12 weeks	↓ co-occurring behaviour symptoms in youth with depression.
Gabbay et al., 2018 [87]	2:1 ratio of EPA to DHA: Initial dose of 1.2 g/day. Doses were raised in increments of 0.6 g/day every 2 weeks (maximum possible dose of 3.6 g/day, combined EPA 2.4 g + DHA 1.2 g)	51 psychotropic medication-free adolescents with MDD aged 12–19 years	10 weeks	n-3PUFAs do not appear to be superior to placebo.
Jahangard et al., 2018 [89]	n-3 PUFAs (1000 mg/day) + sertraline (50–200 mg/day)	50 MDD patients	12 weeks	↓ depression, anxiety, sleep and patients’ competencies to regulate their emotions.
Tayama et al., 2019 [90]	DHA 500 mg/day + EPA 1000 mg/day	20 patients with mild to moderate depression	12 weeks	no significant differences

Abbreviations: EPA = eicosapentaenoic acid; DHA = docosahexaenoic acid; ethyl-EPA = ethyl-eicosapentaenoic acid; AA: Arachidonic acid; CDRS = Childhood Depression Rating Scale; CDI = Childhood Depression Inventory; EPDS = Edinburgh Postnatal Depression Scale; GDS = Geriatric Depression Scale; ↓ = decrease of; ↑ = increase of; MDD: Major depressive disorder; HDRS: Hamilton Depression Rating Scale; BDI: Beck Depression Inventory; PDSS = Postpartum Depression Screening Scale; PEP= Individual-Family Psychoeducational Psychotherapy.

**Table 3:** Double-blind controlled trials of PUFAs in the treatment of bipolar disorders (depressive and manic episodes).

Study	Drugs and Dose	Sample	Treatment Duration	Results
Hirashima et al., 2004 [109]	High dose: EPA, 5.0–5.2 g/day; DHA, 3.0–3.4 g/day; other, 0.3–1.7 g/day	21 patients	4 weeks	no significant differences
Chiu et al., 2005 [107]	valproate 2 g/day and 4.4g/day EPA + 2.4 g/day DHA	16 newly hospitalised patients in the acute manic phase of bipolar disorder	4 weeks	no significant differences
Keck et al., 2006 [110]	EPA 6 g/day in addition to at least one mood stabilizer	121 patients with bipolar depression or rapid cycling bipolar disorder	4 months	no significant differences
Frangou et al., 2006 [111]	ethyl-EPA 1 or 2 g/day added to stable psychotropic medications	75 patients	12 weeks	↓ depressive symptoms measured with HDRS
Murphy et al., 2012 [112]	omega-3 fatty acids plus cytidine, omega-3 fatty acid plus placebo or only placebo in addition to a mood stabilizer	45 patients with type I bipolar disorder	4 months	no benefits of omega-3 fatty acids on affective symptoms
Stoll et al., 1999 [113]	EPA 6.2 g/day + DHA 3.4 g/day	30 patients	16 weeks	↓ depressive symptoms measured with HDRS
Gracious et al., 2010 [114]	ALA in addition to psychotropic medication	children and adolescent with bipolar I or II disorder	16 weeks	significant improvement of overall symptom severity compared with placebo

Abbreviations: EPA = eicosapentaenoic acid; DHA = docosahexaenoic acid; ethyl-EPA = ethyl-eicosapentaenoic acid; ALA = α-linoleic acid; ↓ = decrease of; ↑ = increase of; HDRS: Hamilton Depression Rating Scale.

**Table 4:** Double-blind controlled trials of PUFAs in the treatment of post-traumatic stress disorder (PTSD), obsessive-compulsive disorder (OCD) and substance use disorder.

PTSD				
Study	Drugs and Dose	Sample	Treatment Duration	Results
Matsuoka et al., 2015 [125]	DHA 1.47 g/day + EPA 0.147 g/day	110 patients	12 weeks	not superior to placebo for the secondary prevention of PTSD symptoms
Matsuoka et al., 2016 [126]	DHA 1.47 g/day + EPA 0.147 g/day	110 patients	12 weeks	↑ erythrocyte level of EPA associated with ↓ PTSD symptoms
Matsumura et al., 2017 [127]	DHA 1.47 g/day and EPA 0.147 g/day	83 patients	12 weeks	effective for the secondary prevention of psychophysiological symptoms of PTSD
**OCD**				
Study	Drugs and Dose	Sample	Treatment Duration	Results
Fux et al., 2004 [124]	EPA 2 g/day + stable dose of SSRIs	11 patients with OCD	6 weeks	no effect on anxious, obsessive-compulsive and depressive symptoms
**Substance use disorder**				
Study	Drugs and Dose	Sample	Treatment Duration	Results
Buydens-Branchey & Barnchey, 2006 [122]	EPA + DHA at high dose (3 g/day)	13 patients with substance abuse	12 weeks	↓ anxious symptoms
Buydens-Branchey et al., 2008 [128]	EPA + DHA at high dose (3 g/day)	22 patients with substance abuse	12 weeks	↓ anger and anxiety levels

Abbreviations: EPA = eicosapentaenoic acid; DHA = docosahexaenoic acid; ↓ = decrease of; ↑ = increase of.

**Table 5:** Double-blind controlled trials of PUFAs in the treatment of attention deficit hyperactivity disorder (ADHD).

Study	Drugs and Dose	Sample	Treatment Duration	Results
Voigt et al., 2001 [143]	DHA 0.345 g/day versus placebo. With ADHD medication	63 children (6–12 years old) with ADHD	4 months	no statistically significant improvement in any ADHD symptoms compared to placebo
Richardson et al., 2002 [144]	EPA 0.186 g/day + DHA 0.480 g/day + linolenic acid 0.864 g/day + arachidonic acid 0.042 g/day versus placebo	41 children with ADHD-like symptoms	12 weeks	improvement of cognitive problems and general behaviour in the group treated with PUFAs than placebo
Stevens et al., 2003 [145]	DHA 0.48 g/day + EPA 0.08 g/day + arachidonic acid 0.04 g/day + gamma-linolenic acid 0.096 g/day versus placebo. No ADHD medications	50 children with ADHD-like symptoms	4 months	no significant differences
Hirayama et al., 2004 [146]	EPA 0.1 g/day + DHA 0.5 g/day versus placebo Mostly without ADHD medications (only six subjects had been under medications)	40 children with ADHD	2 months	no evidence of efficacy of omega-3 fatty acids compared to placebo
Sinn and Bryan., 2008 [147]	EPA 93 mg/day + DHA 29 mg/day + gamma-linolenic acid 10 mg/day versus placebo. No ADHD medications	132 children (7 to 12 years) with ADHD		improved in inattention, hyperactivity and impulsivity in most ADHD scales in parents reports; no improvement in teachers reports Limits: No ADHD diagnosis (reported ADHD symptoms)
Vaisman et al., 2008 [148]	EPA 0.156 g/day + DHA 0.095 g/day or EPA 0.153 g/day + DHA 0.096 g/day or placebo	83 children with ADHD	12 weeks	significantly improved executive functioning
Johnson et al., 2009 [149]	EPA 0.558 g/day + DHA 0.174 g/day + gamma linoleic acid 0.06 g/day versus placebo. Only one patient with ADHD medication	75 children and adolescents 8–18 years old with ADHD	3 months	no evidence of efficacy of omega-3 fatty acids compared to placebo
Bélanger et al. 2009 [150]	EPA 0.02–0.025 g/kg/day + DHA 0.85–0.105 g/kg/day versus placebo. No ADHD medications	26 children	16 weeks	improvement in inattention and global ADHD symptoms only in the first phase of the study (weeks 0 to 15)
Gustafsson et al., 2010 [151]	EPA 0.5 g/day	92 children (7 to 12 years) with ADHD	15 weeks	two ADHD subgroups (oppositional and less hyperactive/impulsive children) improved symptoms
Perera et al., 2012 [152]	omega-3 + omega-6 versus placebo. With ADHD medications	98 children (6 to 12 years) with ADHD diagnosis	6 months	improved behavior and learning in restlessness, aggressiveness, completing work and academic performance, but not in inattention, impulsiveness and cooperation with parents and teachers
Manor et al., 2012 [153]	0.3 g of PS and 0.120 g of EPA + DHA (EPA/DHA ratio of 2:1)	200 children with ADHD	15 weeks	improved ADHD symptoms
Milte et al., 2012 [154]	EPA-rich oil (providing EPA 1.109 g/day and DHA 0.108 g/day, DHA-rich oil (providing EPA 0.264 g/day and DHA 1.032 g/day) versus an omega-6 PUFAs oil. No ADHD medications	90 children (7 to 12 years old) with ADHD	4 months	no statistically significant differences between the two groups
Widenhorn-Müller et al., 2014 [155]	EPA 0.6 g/day + DHA 0.120 g/day. No ADHD medications	95 children (6 to 12 years) with ADHD	16 weeks	improved working memory function, but no effects on other cognitive measures or behavioural symptoms in the study population
Bos et al., 2015 [156]	EPA 0.65 g/day + DHA 0.65 g/day	40 young boys (8 to14 years ) with ADHD	16 weeks	↓ symptoms of ADHD, both for individuals with ADHD and typically developing children.

Abbreviations: EPA = eicosapentaenoic acid; DHA = docosahexaenoic acid; PS = phosphatidylserine; ↓ decrease of.

**Table 6:** Double-blind controlled trials of PUFAs in the treatment of autism spectrum disorders.

Study	Drugs and Dose	Sample	Treatment Duration	Results
Amminger et al., 2007 [165]	EPA 0.84 /day + DHA 0.7 g/day	13 children (aged 5 to 17 years) with autistic disorders accompanied by severe tantrums, aggression or self-injurious behavior	6 weeks	improvement of hyperactivity and stereotypy
Bent et al., 2011 [166]	1.3 g/day of omega-3 fatty acids (and 1.1 g of DHA + EPA)	27 children (aged 3 to 8 years) with autism spectrum disorder	12 weeks	improvement of hyperactivity
Bent et al., 2014 [167]	1.3 g/day of omega-3 fatty acids (and 1.1 g of DHA + EPA)	57 children with autism spectrum disorder	6 weeks	improvement of hyperactivity
Yui et al., 2012 [168]	large doses of ARA (40 mg/day) added to DHA (40 mg/day)	13 patients with autism spectrum disorder	16 weeks	improved impaired social interaction by up-regulating signal transduction.
Voigt et al., 2014 [169]	DHA 0.2 g/day or placebo	48 children (3 to 10 years) with autism	6 months	no improvement core symptoms of autism
Mankad et al., 2015 [170]	from 0.75 to 1.5 g/day of EPA + DHA	38 children (2 to 5 years) with autism spectrum disorder	6 months	no significant differences
Sheppard et al., 2017 [171]	Daily doses of Omega-3-6-9 Junior treatment (including 338 mg EPA, 225 mg DHA, 83 mg GLA and 306 mg total omega-9 fatty acids)	31 children 18–38 months of age born at ≤29 weeks of gestation	3 months	evidence of efficacy of omega-3 and -6 fatty acid supplementation in improving aspects of early language development in children at risk for ASD
Mazaheri et al., 2019 [172]	vitamin D (2000 IU/day, VID), omega-3 LCPUFA (722 mg/day DHA, OM) or both (2000 IU/day vitamin D + 722 mg/day DHA, VIDOM)	117 children (2,5 to 8 years) with autism spectrum disorder	12 months	vitamin D and omega-3 LCPUFA reduced irritability symptoms; vitamin D also reduced hyperactivity symptoms

Abbreviations: EPA = eicosapentaenoic acid; DHA = docosahexaenoic acid VID = vitamin D; OM = omega-3; VIDOM = vitamin D + omega-3; ARA = arachidonic acid.

**Table 7:** Double-blind controlled trials of PUFAs in the treatment of anorexia nervosa.

Study	Drugs and Dose	Sample	Treatment Duration	Results
Ayton et al., 2004 [181]	1 g/day E-EPA in addition to standard treatment	7 young patients with anorexia nervosa	3 months	significant growth during E-EPA supplementation
Barbarich et al., 2004 [182]	tryptophan, vitamins, minerals and essential fatty acids (DHA 0.6 g/day and arachadonic acid 0.18 g/day) + fluoxetine	26 patients with anorexia nervosa	6 months	no significant difference in weight gain, anxiety or obsessive-compulsive symptoms
Pirog-Balcerzak et al., 2016 [183]	EPA 0.558 g/day DHA 0.174 g/day + gamma linolenic acid 0.06 g/day, vs. placebo	61 patients with anorexia nervosa	10 weeks	not effective for depressive and compulsive symptoms
Woo et al., 2017 [184]	EPA 300 mg/day + DHA 200 mg/day + standard treatment	21 patients with eating disorders	8 weeks	significant increase in mean percent ideal body weight, but no significant differences in eating disorder, anxiety and depression symptoms
Manos et al., 2018 [185]	EPA 2.12 g/day + DHA 0.6 g/day	24 adolescent females with anorexia nervosa	12 weeks	no significant differences

Abbreviations: EPA = eicosapentaenoic acid; DHA = docosahexaenoic acid.

**Table 8:** Double-blind controlled trials of PUFAs in the treatment of borderline personality disorder.

Study	Drugs and Dose	Sample	Treatment Duration	Results
Zanarini and Frankenburg, 2003 [191]	EPA 1 g/day (with no standard psychiatric therapies)	30 BPD females	8 weeks	↓ aggression, ↓ depression
Hallahan et al., 2007 [192]	EPA 1.2 g/day + DHA 0.9 g/day (added to the standard psychiatric therapies)	49 patients with self-defeating behaviors (39 BPD patients)	12 weeks	↓ depression, ↓ parasuicidal behaviours, ↓ stress reactivity
Bellino et al., 2014 [193]	EPA (1.2 g/day) + DHA (0.6 g/day) in combination with valproic acid (800–1300 mg/day) versus valproic acid (800–1300 mg/day) (plasma range: 50–100 μg/mL)	43 BPD patients	12 weeks	↓ severity of BPDSI, ↓ impulsive behavioural dyscontrol, ↓ anger, ↓ self-mutilating conduct
Gallagher et al., 2017 [194]	n-3 PUFAs including EPA or DHA	40 individuals who self-harmed and 40 controls	10 years	↓ self-harm, ↓depressive symptoms and ↓ impulsivity
Bozzatello et al., 2018 [195]	EPA + DHA + valproic acid	34 patients with borderline personality disorder	24 weeks	↓ outbursts of anger

Abbreviations: EPA = eicosapentaenoic acid; DHA = docosahexaenoic acid; ethyl-EPA = ethyl-eicosapentaenoic acid; BPDSI = borderline personality disorder severity index; ↓ = decrease of; ↑ = increase of.
